# Three-Dimensional Corrective Osteotomy for a Longstanding Multiplanar Fifth Metatarsal Deformity in an Older Adult: A Case Report

**DOI:** 10.7759/cureus.115931

**Published:** 2026-09-08

**Authors:** Yoichi Toyoshima, Akira Matsuoka, Hiroshi Ito, Yuki Saito, Yoshifumi Kudo

**Affiliations:** 1 Orthopedic Surgery, Tokyo Metropolitan Ohtsuka Hospital, Tokyo, JPN; 2 Orthopedic Surgery, Showa University School of Medicine, Tokyo, JPN

**Keywords:** congenital toe deformity, corrective osteotomy, fifth metatarsal deformity, footwear intolerance, multiplanar deformity, three-dimensional assessment

## Abstract

A 75-year-old man presented with a longstanding deformity of the right fifth metatarsal. He had undergone surgery for a congenital toe deformity in early childhood. The fifth metatarsal was shortened and showed lateral displacement, plantar flexion, and external rotation. These deformities resulted in a painful plantar-lateral prominence, callus formation, and difficulty wearing conventional footwear. A three-dimensional corrective osteotomy based on the medial and dorsal translation principles of the long scarfette osteotomy was performed, with an additional asymmetric wedge resection for rotational correction. At the three-year follow-up, bone union had been achieved, the prominence and callus had resolved, and the patient was able to walk without pain and wear conventional footwear comfortably. Residual shortening of the fifth ray caused no functional complaint. This case illustrates a practical approach to the three-dimensional assessment of a complex metatarsal deformity and the correction of each component in the transverse, sagittal, and axial planes.

## Introduction

Congenital toe deformities, including polydactyly, are commonly treated surgically during early childhood, and operative planning depends on the morphology, ray involvement, and associated angular deformity [1-4]. Residual angular deformity, ray malalignment, or abnormal metatarsal morphology may persist after childhood surgery and occasionally require further correction [5-8]. Such deformities are generally addressed during childhood or adolescence; consequently, persistence of an untreated deformity into adulthood is uncommon. Once skeletal maturity has been reached, an established deformity has limited capacity for further remodeling, and correction requires a different approach from that used in children. Although residual deformity after surgery for postaxial polydactyly has been described, reports of symptomatic fifth-ray deformities persisting into older adulthood are limited [5-10]. This report describes the three-dimensional assessment and deformity-specific surgical correction of a longstanding multiplanar fifth metatarsal deformity in an older adult.

## Case presentation

A 75-year-old man reported having undergone surgery at one year of age for a congenital toe deformity that had reportedly been diagnosed as polydactyly. The original medical records and operative details were unavailable. A plantar-lateral prominence over the right fifth metatarsal head had been present since childhood. Pressure from conventional footwear caused pain, and he routinely modified his shoes to avoid contact with the prominence. He had not previously received specialist treatment for the deformity and was referred to our hospital by his family physician. His medical history included well-controlled diabetes mellitus.

On physical examination, the fifth toe demonstrated valgus alignment, and the fifth metatarsal head was prominent on the plantar-lateral aspect of the foot. A callus was present over the plantar-lateral aspect of the fifth metatarsophalangeal joint (Figures 1A, 1B). A faint surgical scar was present along the lateral aspect of the fifth toe. The fifth metatarsophalangeal joint remained mobile.

**Figure 1:**
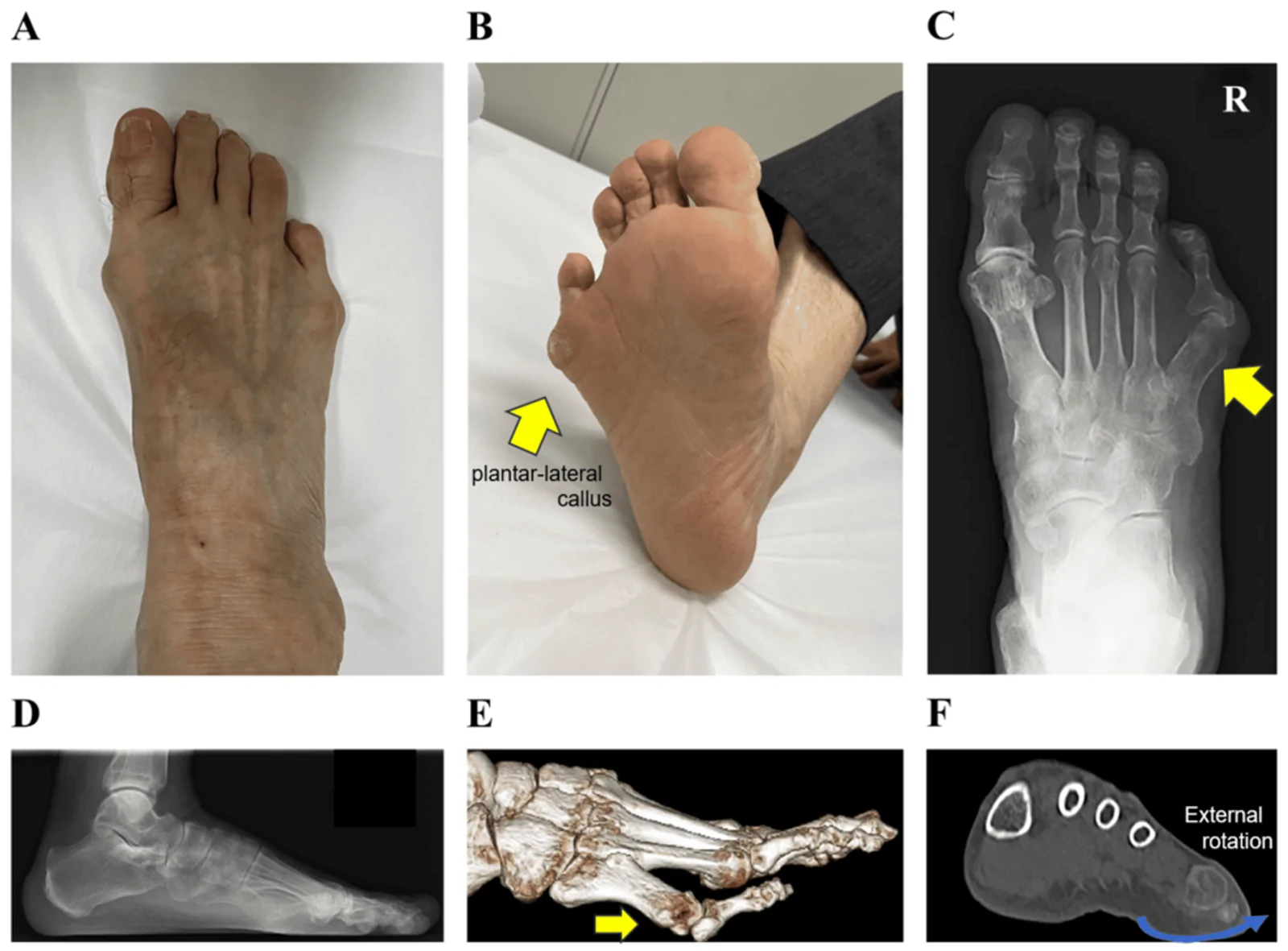
Preoperative clinical photographs and imaging findings. (A, B) Clinical photographs showing valgus alignment of the fifth toe, shortening of the fifth ray, and a plantar-lateral prominence with callus formation. The yellow arrow in (B) indicates the plantar-lateral callus and prominence. (C, D) Anteroposterior and lateral radiographs showing lateral displacement and plantar flexion of the shortened fifth metatarsal. Lateral bony enlargement was present at the base of the proximal phalanx of the fifth toe. The yellow arrow in (C) highlights the lateral displacement of the fifth ray. (E, F) Three-dimensional computed tomography images showing lateral displacement, plantar flexion, and external rotation of the fifth metatarsal. The yellow arrow in (E) indicates the plantar-flexion deformity, and the blue curved arrow in (F) indicates external rotation.

Imaging findings

Plain radiographs and computed tomography demonstrated a shortened fifth metatarsal with lateral displacement in the transverse plane, plantar flexion in the sagittal plane, and external rotation in the axial plane (Figures 1C-1F). Lateral bony enlargement was present at the base of the proximal phalanx of the fifth toe. The bases of the fourth and fifth metatarsals were fused. The fifth metatarsal head was located 19 mm proximal to the fourth metatarsal head. The angle between the longitudinal axes of the fifth metatarsal and proximal phalanx was 42°, and the fourth-fifth intermetatarsal angle was 27°. Given that the pain and footwear intolerance had persisted despite shoe modification, surgical correction was selected.

Surgical technique

A three-dimensional corrective osteotomy was planned using the medial and dorsal translation principles of the long scarfette osteotomy, with an additional asymmetric wedge resection for rotational correction. The long scarfette configuration permits medial and dorsal displacement of the distal fifth metatarsal fragment [11]. A long oblique osteotomy was made from the distal-dorsal aspect of the fifth metatarsal shaft, immediately proximal to the articular surface, toward the proximal- plantar aspect (Figure 2A). The distal fragment was first translated medially along the osteotomy plane to correct the lateral displacement in the transverse plane.

**Figure 2:**
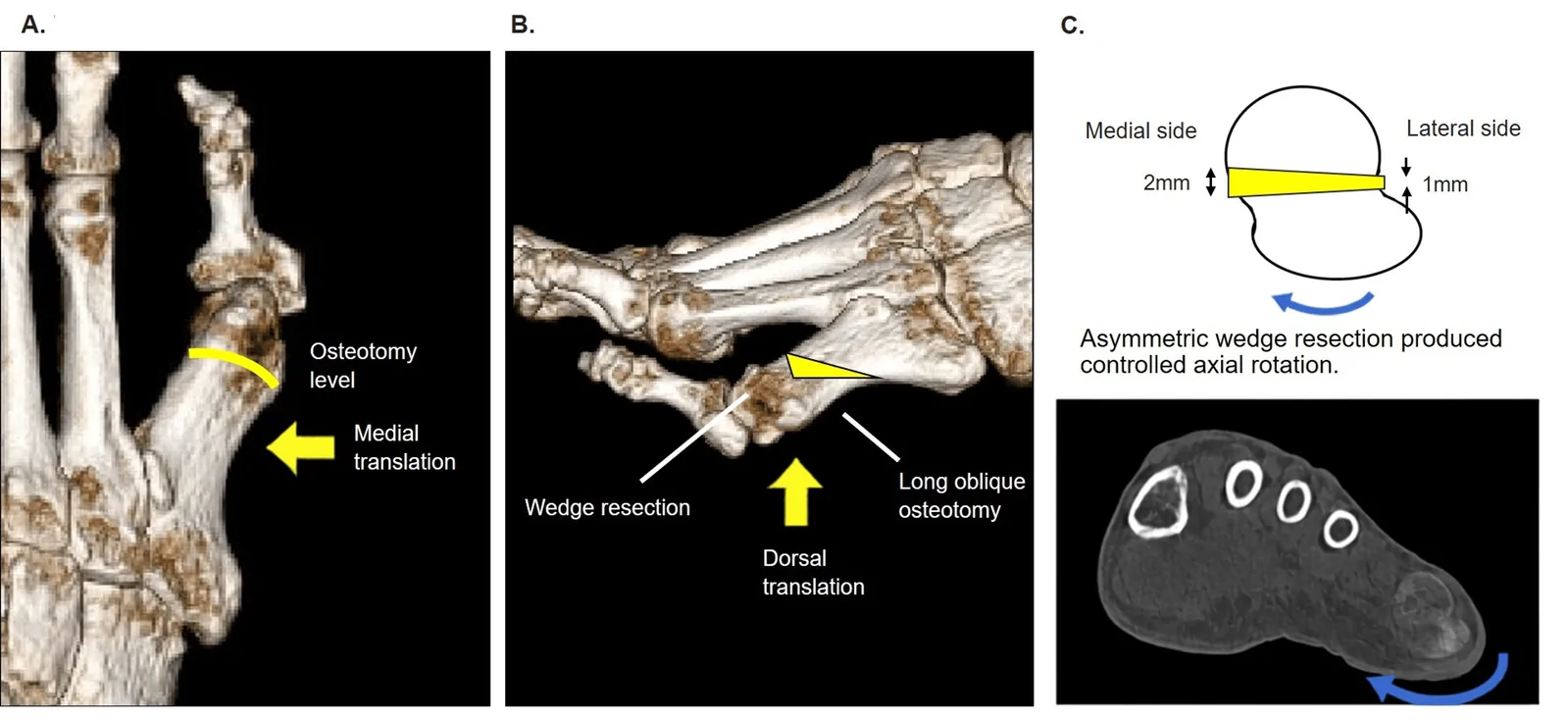
Three-dimensional corrective osteotomy of the fifth metatarsal. (A) Transverse plane correction: A long oblique osteotomy based on the medial translation principle of the long scarfette osteotomy allowed correction of the lateral displacement in the transverse plane. The yellow curved line indicates the osteotomy level, and the yellow arrow indicates medial translation of the distal fragment. (B) Sagittal-plane correction: Wedge resection allowed dorsal translation of the distal fragment and correction of the plantar-flexion deformity. The yellow wedge indicates the resected bone, and the yellow arrow indicates dorsal translation. (C) Axial rotational correction: An additional asymmetric wedge resection, with resection widths of approximately 2 mm on the medial side and 1 mm on the lateral side, produced controlled rotation of the distal fragment and corrected the external rotational deformity in the axial plane. The blue curved arrows indicate controlled axial rotation, and the schematic labels show the asymmetric resection widths on the medial and lateral sides. The figure was assembled using Microsoft PowerPoint (Microsoft Corporation, Redmond, WA).

To correct the plantar-flexion deformity, a wedge-shaped portion was removed from the distal end of the proximal fragment (Figure 2B), allowing dorsal translation of the distal fragment. The procedure was further modified to address the rotational deformity. An asymmetric wedge was resected, measuring approximately 2 mm on the medial side and 1 mm on the lateral side. Closure of the osteotomy after this asymmetric wedge resection produced controlled rotation of the distal fragment and corrected the external rotational deformity in the axial plane (Figure 2C). This modification allowed correction of the transverse, sagittal, and axial components at a single osteotomy site. After fluoroscopic confirmation of alignment, the osteotomy was stabilized with a reconstruction plate, and a lag screw was inserted through the plate to achieve compression. The lateral bony enlargement at the base of the proximal phalanx was also resected to improve alignment of the fifth metatarsophalangeal joint. No additional extensor tendon procedure was required after bony correction. Lengthening of the fifth ray was not attempted because it would have reduced the bony contact area at the osteotomy site and potentially compromised fixation and union.

Postoperative course

Postoperatively, the foot was protected in an orthosis, and non-weight-bearing was maintained for four weeks. Partial weight-bearing was initiated at four weeks. Full weight-bearing was permitted after radiographic healing had been confirmed. Bone union was confirmed three months postoperatively. At the three-year follow-up, the corrected alignment was maintained without implant-related complications. Postoperative radiographic measurements demonstrated substantial angular correction, although shortening of the fifth ray remained. The fifth metatarsal head was located 17 mm proximal to the fourth metatarsal head, compared with 19 mm preoperatively. The angle between the longitudinal axes of the fifth metatarsal and proximal phalanx improved from 42° to 2°, and the fourth-fifth intermetatarsal angle improved from 27° to 6°. The plantar-lateral prominence had been corrected, and the associated callus had completely resolved (Figures 3A-3E). The patient reported no pain during walking and was able to wear conventional footwear without modification or discomfort. He also reported satisfaction with the surgical outcome. Although shortening of the fifth ray remained, it caused no functional complaint. The American Orthopaedic Foot & Ankle Society lesser toe metatarsophalangeal-interphalangeal (MTP-IP) score improved from 40 points preoperatively (pain, 20; function, 20; alignment, 0) to 100 points at the final follow-up [12].

**Figure 3:**
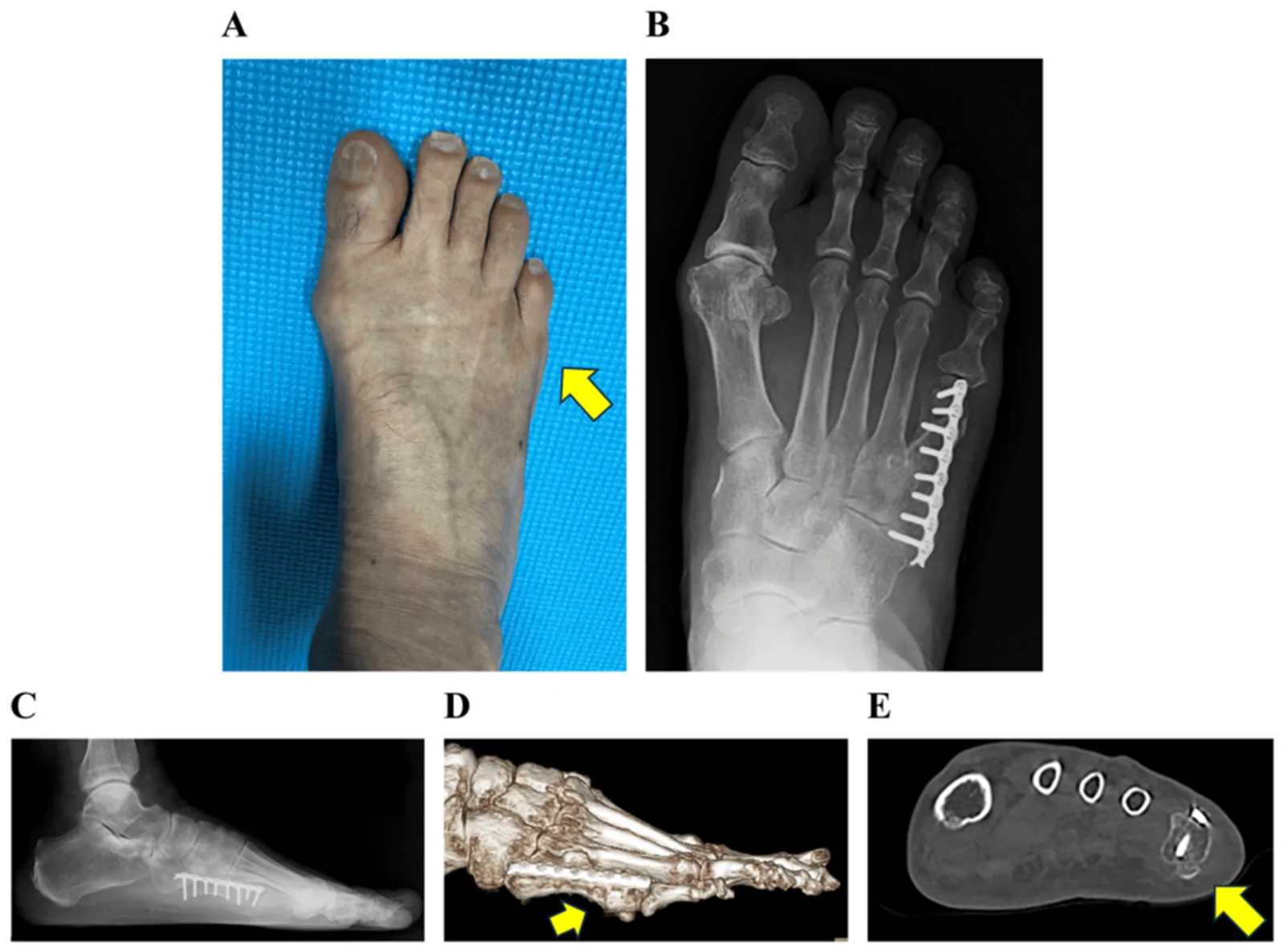
Postoperative clinical photographs, radiographs, and three-dimensional computed tomography images. (A) The plantar-lateral prominence and valgus alignment were corrected, although shortening of the fifth ray remained. The yellow arrow indicates the site of the corrected plantar-lateral prominence. (B, C) Postoperative radiographs showing improved alignment of the fifth metatarsal, with the fifth metatarsal axis approximately parallel to that of the fourth metatarsal. The plantar-flexion deformity was corrected on the lateral view. (D, E) Three-dimensional computed tomography images showing correction of the lateral displacement, plantar flexion, and external rotational deformity. The yellow arrow in (D) highlights correction of the plantar-flexion deformity, and the yellow arrow in (E) highlights correction of the external rotational deformity. Bone union was achieved at the osteotomy site.

## Discussion

This case involved a longstanding multiplanar deformity of the fifth metatarsal that remained untreated for more than seven decades and ultimately caused pain, plantar-lateral callus formation, and marked difficulty with conventional footwear. The deformity consisted of lateral displacement in the transverse plane, plantar flexion in the sagittal plane, external rotation in the axial plane, and shortening of the fifth ray. Residual deformities after childhood treatment may include angular malalignment and abnormal fifth-ray morphology [5-8]. Once skeletal maturity has been reached, symptomatic established deformities may require direct bony correction rather than reliance on further remodeling. Surgical treatment should therefore be planned according to the individual components of the deformity rather than treating the condition as a simple lateral prominence [5, 6].

The precise cause of the deformity remains uncertain because the original diagnosis, preoperative anatomy, and operative records from the childhood surgery were unavailable. The only available information was the patient’s recollection that the original diagnosis had been polydactyly. Plausible explanations include residual deformity after accessory-toe excision, a growth disturbance related to the childhood procedure, or an underlying congenital abnormality of the fifth metatarsal [5-10]. Previous studies have shown that postoperative alignment after treatment of postaxial polydactyly may be influenced by the selected excised ray, the extent of metatarsal involvement, and reconstruction of the intermetatarsal soft tissues [6-10]. However, the available findings were insufficient to reconstruct the original deformity or determine the details of the childhood procedure. Accordingly, this report focuses on the analysis and correction of the established adult deformity rather than on determining its exact etiology.

Conventional procedures for bunionette deformity primarily address lateral prominence or transverse-plane deviation using condylar resection, translation osteotomy, reverse Ludloff osteotomy, or long oblique diaphyseal osteotomy [11,13-16]. The long scarfette configuration permits medial and dorsal translation of the distal fifth metatarsal fragment and is therefore useful when transverse- and sagittal-plane correction is required [11]. The reverse Ludloff osteotomy has also been described for bunionette correction, particularly when substantial translation is required [14]. The present deformity, however, also required targeted correction of plantar flexion and external rotation. In the present case, medial translation corrected the lateral displacement, and selective wedge resection permitted dorsal translation and correction of plantar flexion. However, these maneuvers alone were insufficient to correct the external rotational deformity. An additional asymmetric wedge resection, wider medially than laterally, was therefore used to generate controlled axial rotation of the distal fragment. This modification allowed the transverse, sagittal, and axial components to be corrected at a single osteotomy site while preserving sufficient bony contact for stable fixation and union.

The procedure achieved radiographic union, correction of the plantar-lateral prominence, resolution of the callus, complete relief of walking pain, and restoration of comfortable footwear use. Although shortening of the fifth ray remained, it caused no functional complaint. Fifth-ray lengthening was deliberately avoided because it would have reduced the osseous contact area at the osteotomy site and potentially compromised fixation and union. Given the region-specific intraosseous vascular anatomy of the fifth metatarsal [17], preservation of local vascularity and adequate bony contact was considered important. The operative principles of this technique may be reproducible in similar multiplanar fifth metatarsal deformities when each deformity component is clearly identified preoperatively. However, the amount and direction of translation and wedge resection should be tailored to the individual anatomy and severity of the deformity. The principal limitations are the uncertainty regarding the original diagnosis and childhood procedure and the three-year follow-up period. Therefore, the generalizability of this technique cannot be established from this case alone. Nevertheless, this case suggests the potential value of a deformity-specific approach in which each transverse-, sagittal-, and axial-plane component is identified preoperatively and matched to a corresponding corrective maneuver.

## Conclusions

A longstanding multiplanar fifth metatarsal deformity in an older adult was successfully treated with a three-dimensional corrective osteotomy that addressed the transverse, sagittal, and axial components. The procedure resulted in bone union, pain relief, resolution of callus formation, and improved footwear tolerance. This case illustrates the potential value of a three-dimensional, deformity-specific approach when conventional single-plane correction is insufficient for a complex fifth metatarsal deformity.
